# Drug-eluting contact lens containing cyclosporine-loaded cholesterol-hyaluronate micelles for dry eye syndrome†

**DOI:** 10.1039/c9ra02858g

**Published:** 2019-05-28

**Authors:** Jonghwan Mun, Jee won Mok, Sanghoon Jeong, Seonghwi Cho, Choun-Ki Joo, Sei Kwang Hahn

**Affiliations:** Department of Materials Science and Engineering, Pohang University of Science and Technology (POSTECH) 77 Cheongam-ro, Nam-gu Pohang Gyeongbuk 37673 Korea skhanb@postech.ac.kr +82 54 279 2399 +82 54 279 2159; Department of Ophthalmology and Visual Science, Seoul St. Mary's Hospital, Collage of Medicine, The Catholic University of Korea 505, Banpo-dong Seocho-gu Seoul 06591 Korea ckjoo@catholic.ac.kr +82 2 533 3801 +82 2 2258 1173

## Abstract

A contact lens is an attractive tool for the delivery of ophthalmic drugs, but it has several issues such as the burst release of drugs and the limited drug loading capacity. To overcome these limitations, we developed a cholesterol-hyaluronate (C-HA) micelle-embedded contact lens for efficient hydrophobic drug loading and long-term controlled drug delivery. The contact lens was fabricated *via* photopolymerization of hydroxyethyl methacrylate (HEMA) using ethylene glycol dimethacrylate (EGDMA) as a cross-linker. The C-HA micelle-loaded contact lens showed statistically significant improvement in wettability and mechanical strength, maintaining the optical transmittance. *In vitro* drug release tests revealed the controlled delivery of cyclosporine for more than 12 days. Furthermore, the Schirmer tear test, corneal fluorescein staining, and MMP9 fluorescein analysis confirmed its therapeutic effect on dry eye syndrome in disease model rabbits.

## Abstract 2

A cholesterol-hyaluronate (C-HA) micelle embedded contact lens was developed for efficient hydrophobic drug loading and long-term controlled drug delivery.

## Graphical abstract

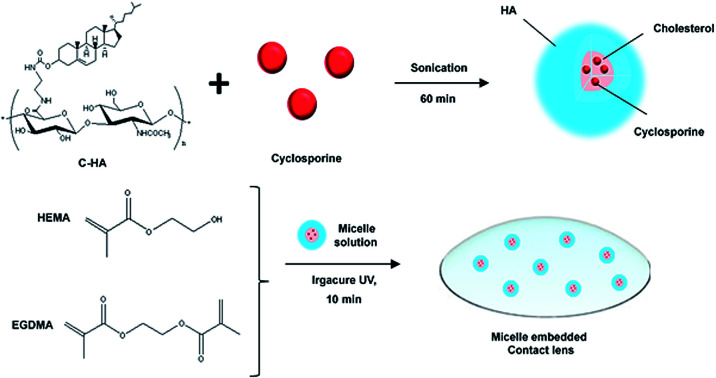

## 1. Introduction

Eye diseases are commonly treated by periodic eye drops of relevant drugs due to patient compliance. In this case, however, the drug delivery efficiency is reported to be less than 1% with significant drug loss.^1,2^ In addition, the residence time of drugs delivered by eye drops is in the range of 1–3 min in the tear film with low bioavailability.^3–5^ To overcome these problems, drugs are delivered at a high concentration or injected at the target site, which reduces patient compliance and causes other side effects.^6^ Contact lenses have been widely used for vision correction, eye protection, and aesthetic applications. Since contact lenses are directly placed on the cornea, they can be an ideal system for delivering drugs to the anterior chamber. For example, glaucoma drug delivery *via* contact lenses showed 10 times higher efficiency than that by eye drops.^7^ In addition, drugs can be continuously delivered into the eye through a contact lens rather than being temporarily delivered by eye drops.^8^

A variety of nanoparticles have been developed for long-term controlled drug delivery such as polymeric micelles, liposomes, and microemulsions.^9–12^ In particular, polymeric micelles have attracted great attention due to their efficient hydrophobic drug loading and controlled drug delivery.^13–15^ Hydrophobic drugs have been encapsulated in amphiphilic block copolymers or conjugated to the hydrophilic polymers, forming a micelle structure.^16–18^ Hyaluronate (HA) is known to be hygroscopic and maintains a high water content *via* chain–chain interactions.^19^ The superior biocompatibility of HA can provide comfort to the eye with a high water content.^20^ In addition, HA derivatives have been used to increase the bioavailability and the ocular residence time of ophthalmic drugs.^21,22^

**Fig. 1:**
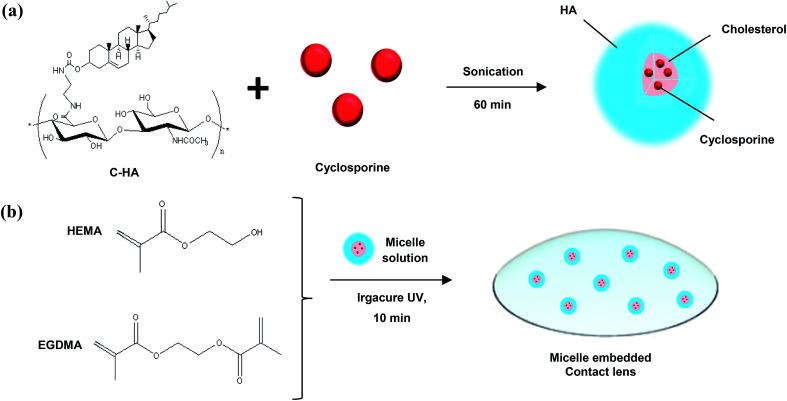
Schematic illustrations for (a) the preparation of cyclosporine-loaded C-HA micelles and (b) the fabrication of the micelle-embedded contact lens.

In this work, we developed a drug-eluting contact lens containing cyclosporine-loaded cholesterol-HA (C-HA) micelles for the treatment of dry eye syndrome. Fig. 1a shows a schematic for the preparation of the cyclosporine-loaded C-HA micelles and Fig. 1b shows the fabrication of a C-HA micelle-embedded contact lens. Cyclosporine is a hydrophobic drug for keratoconjunctivitis sicca (dry eyes), and it was encapsulated in C-HA micelles for the treatment of dry eye syndrome. The cyclosporine-loaded C-HA micelles were characterized by dynamic light scattering (DLS), zeta potential analysis, transmission electron microscopy (TEM), and high-performance liquid chromatography (HPLC). In addition, we assessed the mechanical properties and wettability of the C-HA micelle-embedded contact lenses. After *in vitro* drug release tests, the Schirmer tear test, corneal fluorescein staining, and fluorescein analysis of metallopeptidase 9 (MMP9) were performed to assess the therapeutic effect of the drug-eluting contact lens on dry eye disease (DED) model rabbits.

## 2. Experimental section

### 2.1 Materials

Sodium hyaluronate (HA, MW 100 kDa) was purchased from Lifecore Biomedical (Chaska, MN). Tetrabutylammonium (TBA)-OH was obtained from Alfa Aesar (Ward Hill, MA). Dowex resin, ethylenediamine, cholesteryl chloroformate, hydroxyethyl methacrylate (HEMA), ethylene glycol dimethacrylate (EGDMA), cyclosporine, insulin, epithelial growth factor (EGF), hydrocortisone, and chleratoxin were obtained from Sigma-Aldrich (St. Louis, MO). Dulbecco's Modified Eagle's Medium/F-12 Nutrient Mixture Ham (DMEM/F-12) mixture (3/1), penicillin and streptomycin were obtained from WelGENE (Seoul, Korea).

### 2.2 Preparation methods

#### 2.2.1 Synthesis of cholesterol-hyaluronate

HA was modified with tetrabutylammonium salt to prepare HA-TBA, as reported elsewhere.^23,24^ Briefly, a cation exchange resin of DOWEX was mixed with TBA-OH to prepare Dowex-TBA resin. Then, it was reacted with the sodium salt of HA for 3 h. The supernatant was filtered to remove the Dowex resin, obtaining a clear HA-TBA solution, and lyophilized for 3 days. C-HA was synthesized as previously reported elsewhere.^25^ Cholesteryl-2-aminoethylcarbamate (CAEC) was synthesized by mixing cholesteryl chloroformate and ethylenediamine in anhydrous dichloromethane. The mixed solution was stirred on ice for 1 h. The resulting reaction solution was washed with DI water and dried over anhydrous magnesium sulfate. To synthesize C-HA, 100 mg of HA-TBA was dissolved in DMSO (1 wt%) and reacted with 0.33 mL of CAEC dissolved in the mixture of DCM and methanol (1 : 1, v/v). The reaction solution was stirred at room temperature for 30 min, which was mixed with 4.2 mg of 4-(4,6-dimethoxy-1,3,5-triazin-2-yl)-4-methylmorpholinium chloride. After stirring at room temperature for 24 h, the resulting product was dialyzed against a large excess amount of 0.5 M NaCl solution, 50% ethanol, and water and then lyophilized for 3 days. The cholesterol content was determined by proton nuclear magnetic resonance (^1^H NMR) analysis (DPX500, Bruker, Germany) in deuterium oxide.

#### 2.2.2 Cytotoxicity test of C-HA micelles

The cytotoxicity of C-HA in human cornea epithelial cells (HCECs) was assessed by a CCK-8 assay. The cells were suspended in the mixture of DMEM/F-12 (3/1) with 5% of fetal bovine serum (FBS), 5 μg mL^−1^ of insulin, 10 ng mL^−1^ of EGF, 500 ng mL^−1^ of hydrocortisone, 30 ng mL^−1^ of choleratoxin, penicillin and streptomycin and seeded with 100 μL of the cell suspension containing 1.3 × 105 cells per mL on each well of 96-well cell culture plates for 24 h. C-HA with a cholesterol content of 3.5 mol% was dissolved in SFM at the concentrations of 10, 20, 50, 100, 150, 200, and 500 μg mL^−1^ and placed in a 37 °C incubator for 24 h. Then, the samples were washed with PBS thrice and 10 μL of CCK-8 assay solution was added to each well. After incubation for 4 h, the optical density was measured at 480 nm with a microplate reader.

#### 2.2.3 Preparation of cyclosporine/C-HA micelles

C-HA (0.1 wt%) was dissolved in water and cyclosporine was dissolved in DMSO (0.25 wt%). The solutions were mixed and ultra-sonicated for 60 min. The resulting solution was poured into a dialysis membrane (MWCO of 3500 Da) and dialyzed against distilled water for a day to remove the unloaded cyclosporine and DMSO.

#### 2.2.4 Characterization of prepared cyclosporine/C-HA micelles

The hydrodynamic diameter and zeta potential of prepared C-HA micelles in an aqueous solution were measured by DLS (Zetasizer Nano ZS, Malvern Instruments, Worcestershire, UK). The successful formation of cyclosporine/C-HA micelles was assessed by TEM (JEM-1011, JEOL). For TEM analysis, *ca.* 20 μL of the micelle solution was dropped on a TEM grid and then air-dried.

#### 2.2.5 Fabrication of cyclosporine/C-HA micelle-embedded contact lens

The cyclosporine/C-HA micelle solution (80 μL) was mixed with HEMA (320 μL). Then, EGDMA (10 μL) and TPO Irgacure (2.4 mg) were added into the above mixture. The final mixed solution (80 μL) was injected into a contact lens mold and cured at 340 nm under a nitrogen environment for 15 min. After polymerization, the contact lens was soaked in water for hydration. The encapsulation of cyclosporine/C-HA micelles into the PHEMA contact lens was assessed by X-ray photoelectron spectroscopy (XPS).

### 2.3 Characterization methods

#### 2.3.1 Optical transmittance analysis

The optical transmittance of the contact lens hydrogel was analyzed with a UV-Vis spectrometer (S-3100, Scinco). The fabricated contact lenses were placed on the measurement holder and the transmittance was measured at the wavelength of 200–800 nm.

#### 2.3.2 Tensile strength test

The tensile strength of C-HA micelle-embedded contact lenses was measured using Instron 3344 (Instron Corp). The loading rate was 5 mm min^−1^. The specimens had a width of 15 mm and a thickness of 10 mm. The gauge length was 64 mm.

#### 2.3.3 Equilibrium water content analysis

The equilibrium water content (EWC) was calculated by measuring the weights of contact lenses in the dried state (*W*_dry_) and wet state (*W*_wet_). The EWC value was determined by the increased weight of contact lenses during hydration using the following equation:EWC = (*W*_wet_ − *W*_dry_)/*W*_dry_ × 100

#### 2.3.4 Water evaporation test

The water evaporation through the contact lenses was assessed with 1.5 mL of e-tube. One mL of PBS was filled in 1.5 mL of e-tube and the contact lens was glued to the rim of the e-tube. The e-tube was kept tilted so that the PBS could contact the contact lens. Then, the weight of e-tubes was measured every 5 min for a total of 60 min.

#### 2.3.5 Water contact angle measurement

The contact angle of PHEMA contact lenses with and without C-HA micelles was measured with Smartdrop (Femtofab) after dropping 5 μL of water on the contact lenses.

#### 2.3.6 *In vitro* drug loading and release test

The drug loading efficiency of cyclosporine in the C-HA micelles was determined by HPLC analysis. Acetonitrile was added to the micelle solution at a volume ratio of 4/1 to break the micelle structure. HPLC analysis was performed using the following systems: a Waters 1525 binary HPLC pump, a Waters 2487 dual k absorbance detector, a Waters 717 plus autosampler, and a Symmetry™ 300 C18 5 μm column (Waters, MA). The mobile phase was a mixture of acetonitrile and distilled water at a volume ratio of 70/30, and the flow rate was 0.8 mL min^−1^. The column was placed in a column oven set at 80 °C and the detection wavelength was 215 nm for cyclosporine. An *in vitro* drug release test of cyclosporine from the cyclosporine/C-HA micelle-embedded contact lenses was carried out for 288 h. The cyclosporine/C-HA micelle-embedded contact lenses were immersed in 1 mL of PBS using a 24-well plate in an incubator at 37 °C. At the predetermined time intervals, each PBS sample containing the contact lens was obtained and replaced with fresh PBS. The concentration of cyclosporine in the samples was measured by HPLC, as described above.

### 2.4 *In vivo* therapeutic effect test

#### 2.4.1 Preparation of dry eye syndrome model rabbits

All animal studies with New Zealand White rabbits (2–3 kg, Orient Bio, Seoul, Korea) were approved by the Institutional Review Board of Catholic University and performed in accordance with the Association for Research in Vision and Ophthalmology Statement for the Use of Animals in Ophthalmic and Vision Research. The rabbits received 3-concanavalin A injections (Con A, Sigma L7647), one each into the inferior lacrimal gland (ILG), the palpebral portion of the superior lacrimal gland (PSLG), and the orbital portion of the superior lacrimal gland (OSLG). Using a 26-gauge needle, 10 mg of Con A in 1 mL was injected, which was repeated after 1 and 3 days.

#### 2.4.2 Corneal fluorescein staining and analysis

For fluorescein staining, sodium fluorescein was applied to the ocular surface of the animals, typically without sedation. Five min after application, corneal fluorescein staining was scored under a microscope using a blue light. We observed that the irregularities in the eyes, such as abrasion and inflammation, fluoresce with a greater intensity than that of the healthy corneal tissue.

#### 2.4.3 Schirmer tear test

Schirmer strips (EagleVision, Denville, NJ) were inserted into the space between the cornea and the palpebral conjunctiva at the midpoint of the lower eye lid. The tear production was determined based on the length of the moisturized strip measured after 5 min. The reading data were obtained in triplicate and averaged for the analysis. After recording the tear production at 5 min, the strips were left in place for at least 20 mm until wetting.

## 3. Results and discussion

### 3.1 Characteristics of cholesterol-hyaluronate micelles

C-HA was synthesized by the conjugation of HA-TBA with CAEC. The cholesterol content of C-HA was determined by ^1^H NMR analysis (ESI Fig. S1†). The grafted ratio of cholesteryl groups was about 3.8 mol%, which was calculated from the integration ratio between the peaks of the *N*-acetyl group of HA (*δ* = 1.96, COCH_3_) and the methyl group of cholesterol (*δ* = 0.79, CH_3_). C-HA self-assembled into micelle-like nanoparticles in the aqueous solution due to the hydrophobic interaction between cholesterols.^26^ The hydrophobic drug cyclosporine used for dry eye syndrome was loaded in the C-HA micelles by the sonication and dialysis method. The formation of cyclosporine/C-HA micelles and the loading efficiency of cyclosporine within the C-HA micelles were analyzed by DLS and TEM. The diameter was measured to be 290.0 ± 35.95 nm (*n* = 3) and the zeta potential of the particle was measured to be −17.4 ± 3 (*n* = 3) by DLS (ESI Fig. S2a and b†). The TEM image in Fig. S3† shows spherical particles with a diameter of *ca.* 300 nm. As shown in Fig. S4,† there is negligible cytotoxicity in HCECs for the C-HA micelle concentration from 10 to 500 μg mL^−1^.

### 3.2 Characterization of drug-eluting contact lens

#### 3.2.1 Optical transmittance of C-HA micelle-embedded contact lens

**Fig. 2:**
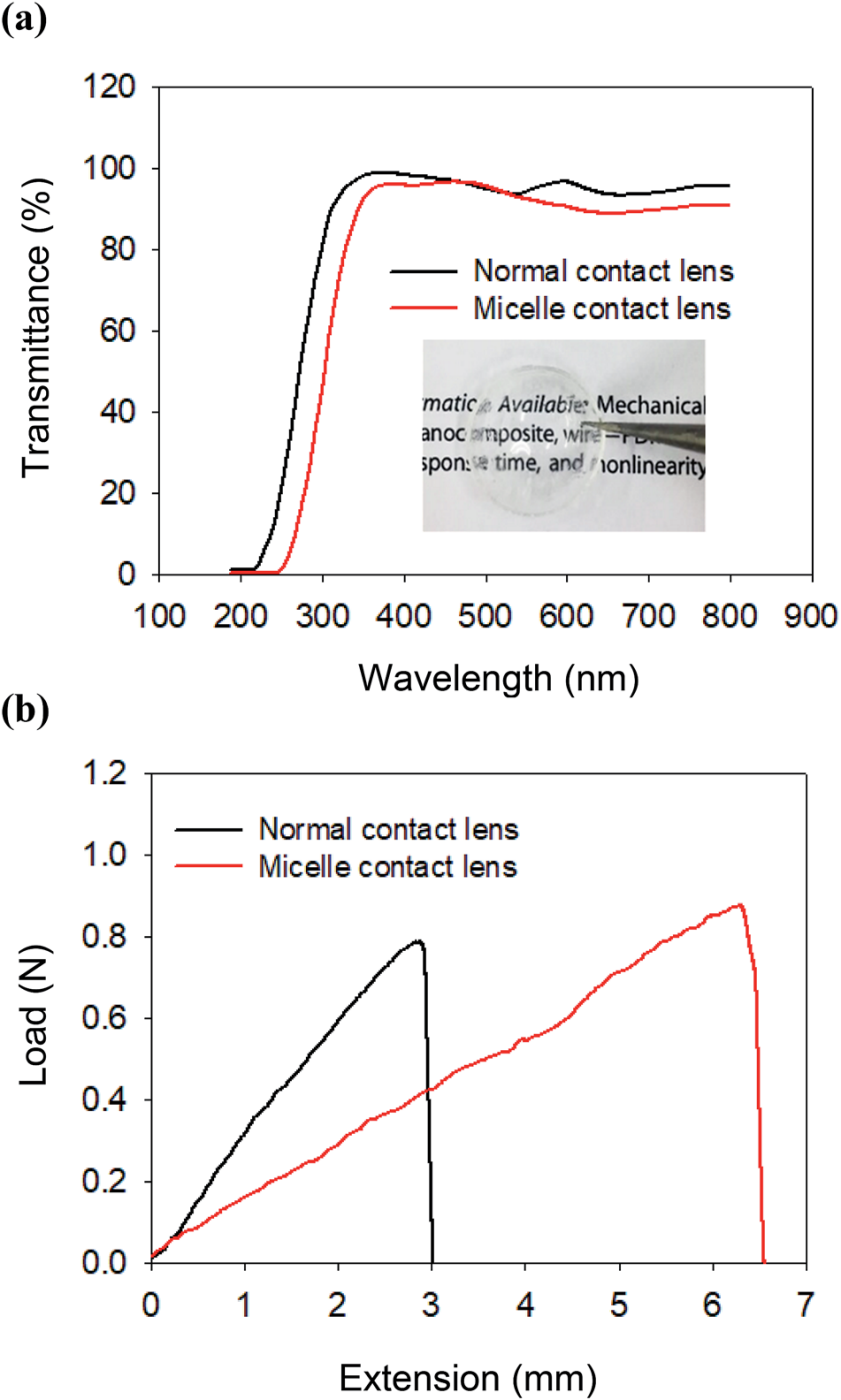
(a) The transmittance spectra of poly(2-hydroxyethyl methacrylate) hydrogel contact lens (control, black) and C-HA micelle-embedded contact lens (red). (b) The tensile tests of normal contact lens (black) and C-HA micelle-embedded contact lens (red).

The successful encapsulation of cyclosporine/C-HA micelles into the contact lens was confirmed by XPS analysis (ESI Fig. S5†). The optical clarity of the micelle-embedded contact lenses was characterized by measuring the transmittance in the range from 200 nm to 800 nm (Fig. 2a). The normal contact lens exhibited *ca.* 95% of transmittance and the C-HA micelle-embedded contact lens showed *ca.* 90% of transmittance. The transparency decreased due to the C-HA micelles, which might not significantly affect visual clarity. The letters under the C-HA micelle-embedded contact lens can be clearly seen without affecting visibility (inset, Fig. 2a).

#### 3.2.2 Tensile strength of C-HA micelle-embedded contact lens

The mechanical properties of contact lens materials are important for long-term durability. Fig. 2b shows the stress–strain curves for the tensile property of the C-HA micelle-embedded contact lens by using Instron. The extension of the C-HA micelle-embedded contact lens was longer than that of the normal contact lens. In other words, the C-HA micelle-embedded PHEMA contact lens was more stretchable than the normal contact lens, reflecting that the C-HA micelle nanoparticles in the contact lens improved the elasticity of the contact lens. In addition, when the tensile specimen began to break, the C-HA micelle-embedded contact lens was slightly extended, resisting the breakage of the hydrogel network.

#### 3.2.3 Equilibrium water content of C-HA micelle-embedded contact lens

**Fig. 3:**
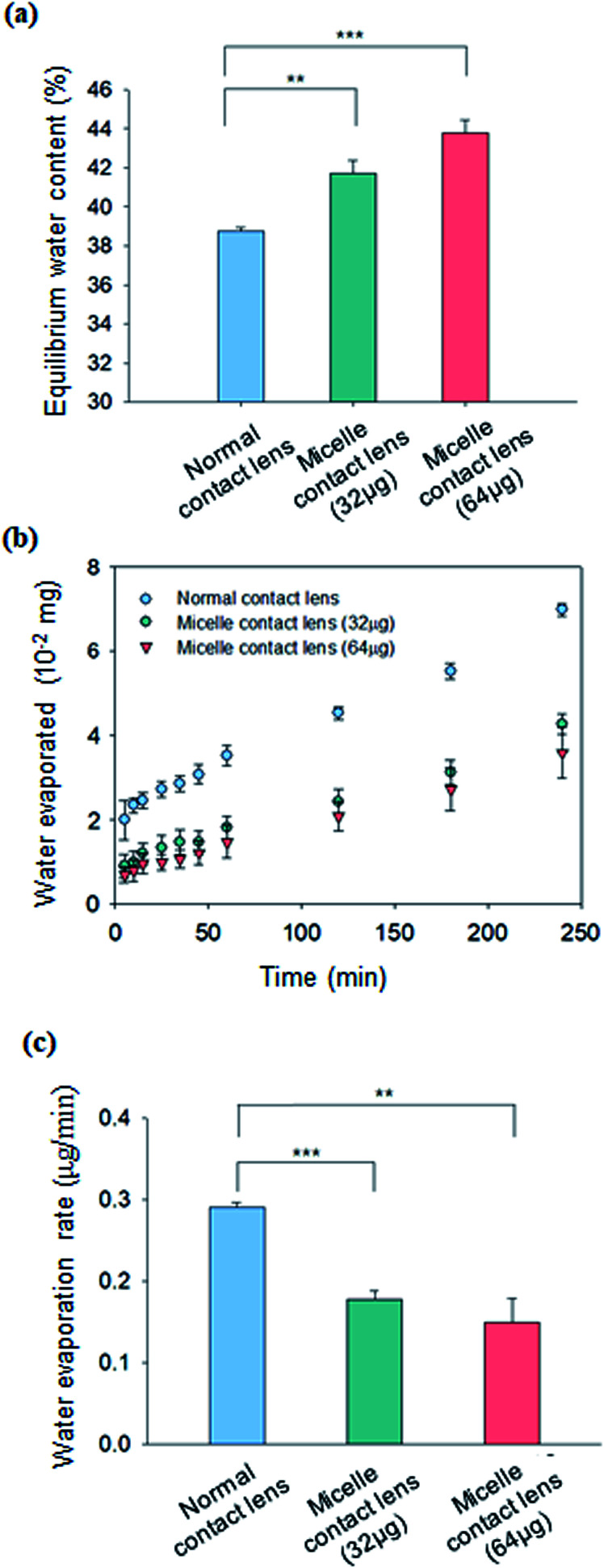
(a) Equilibrium water content of C-HA micelle-embedded contact lens (32 μg of C-HA and 64 μg C-HA micelles) (*n* = 3; **, *P* ≤ 0.01; ***, *P* ≤ 0.0001 *vs*. the control). (b) Water evaporation from C-HA micelle-embedded contact lens and normal contact lens over time (*n* = 3). (c) Water evaporation rate calculated from the slope of water evaporated *vs.* time (*n* = 3; **, *P* ≤ 0.01; ***, *P* ≤ 0.0001 *vs.* the control).

The swelling characteristics of contact lenses are very important parameters for ocular applications. In this regard, the equilibrium water content (EWC) was measured and compared for the C-HA micelle-embedded contact lens and the control normal contact lens. EWC of contact lenses was measured by weighing the contact lenses before and after swelling. Fig. 3a shows the EWC (%) values for the C-HA micelle-embedded contact lens and the control PHEMA contact lens. The water content of the 64 μg C-HA micelle-embedded contact lens was *ca.* 43%, which was higher than *ca.* 39% of the PHEMA contact lens (Fig. 3a). These results indicate that the wettability of the contact lens is drastically enhanced by the hydrophilic HA of the C-HA micelles in the contact lens.

#### 3.2.4 Hygroscopic characteristics of the C-HA micelle-embedded contact lens

The water evaporation test was performed to evaluate the hygroscopic property of contact lenses. The water evaporation rate was determined by measuring the weight change of a 1.5 mL e-tube after sealing with the C-HA micelle-embedded contact lens and the normal contact lens as a control. As shown in Fig. 3b, water evaporating from 1.5 mL of e-tube sealed with the C-HA micelle-embedded contact lens was significantly lower than that of the control contact lens. In Fig. 3c, the water evaporation rate is calculated from the slope of water evaporated *vs.* time. The water evaporation rate was significantly reduced in the C-HA micelle-embedded contact lens (0.149 μg min^−1^) compared to that observed for the control (0.29 μg min^−1^). From the results, we could confirm the improved hygroscopic property of the C-HA micelle-embedded contact lens.

#### 3.2.5 Wettability of the C-HA micelle-embedded contact lens

**Fig. 4:**
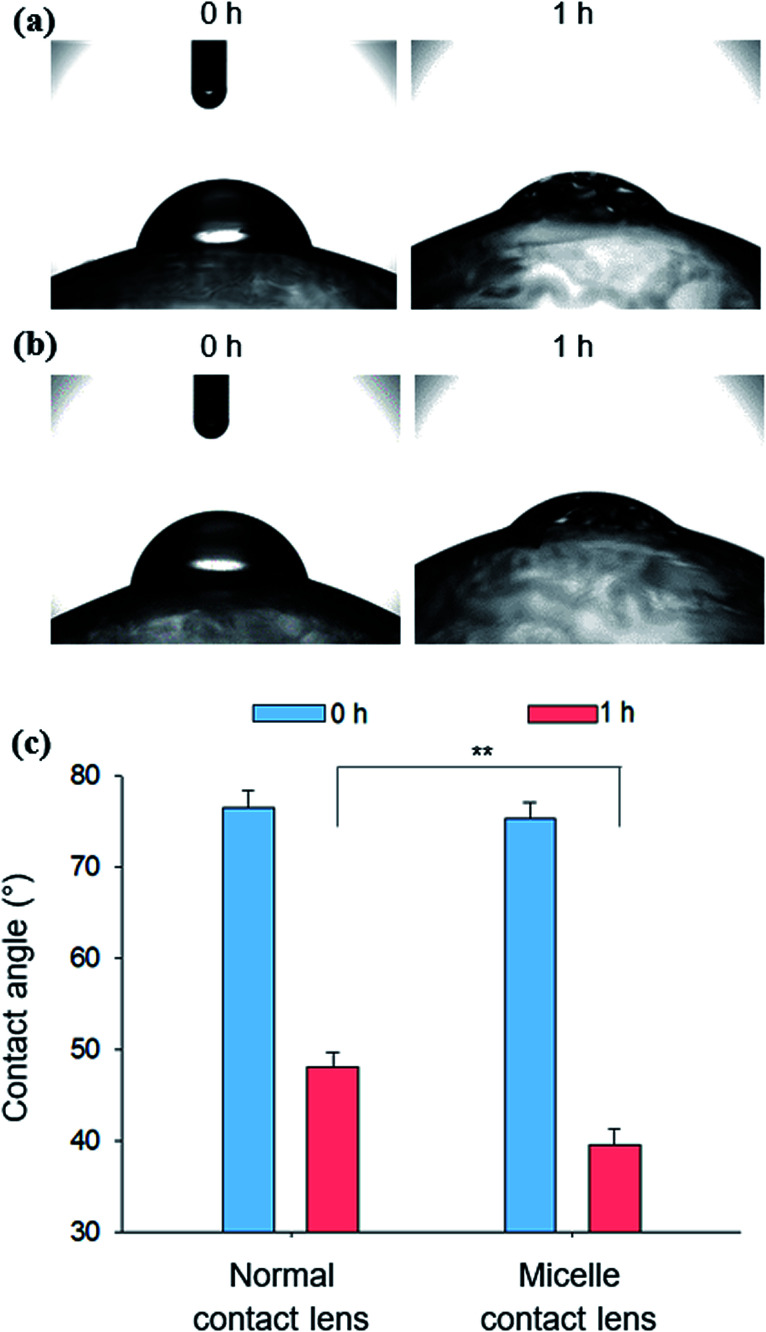
Photographs for the absorption of a water droplet on (a) normal contact lens and (b) C-HA micelle-embedded contact lens. (c) Water contact angles for C-HA micelle-embedded contact lenses and normal contact lenses measured after 0 and 1 h (*n* = 3; **, *P* ≤ 0.01; *vs.* the control).

Water contact angles were measured to determine the wettability of the micelle-embedded contact lens. The contact angles were measured immediately and after 1 h after dropping a 5 μL water droplet on the C-HA micelle-embedded contact lenses (Fig. 4a) and the normal control contact lenses (Fig. 4b). As a result, both groups showed a contact angle of about 77.55° on average just after dropping the 5 μL water droplet. However, after 1 h, the average contact angle was reduced to 39.6° for the C-HA micelle-embedded contact lens and to 48.07° for the control contact lens. The results show that C-HA micelle-embedded contact lenses have higher wettability than the control contact lenses, implying the low adhesion to contaminating lipids.

#### 3.2.6 *In vitro* drug loading and release

**Fig. 5:**
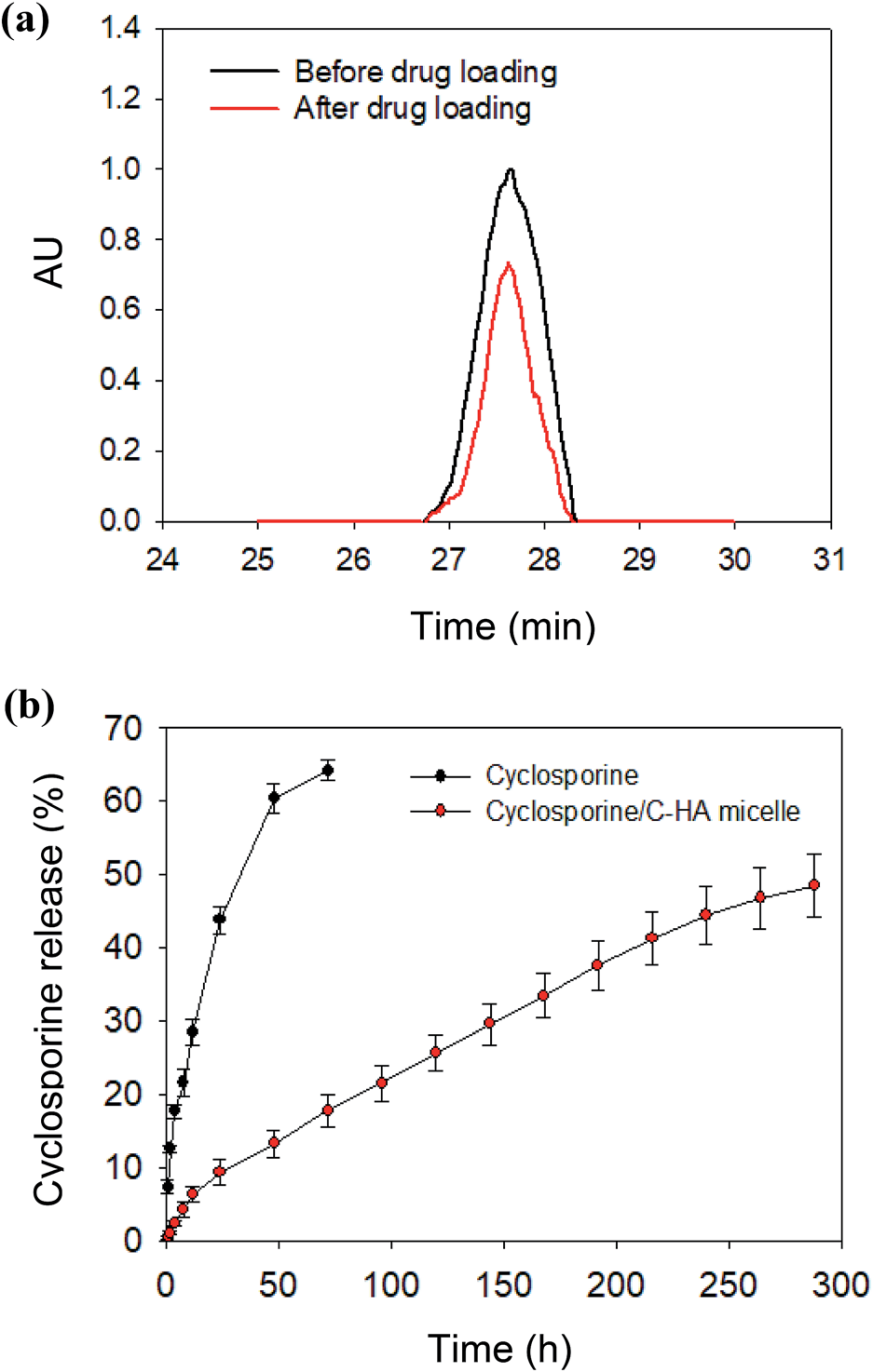
(a) High-performance liquid chromatograms of cyclosporine at 215 nm (black) before and (red) after drug loading to C-HA micelles. (b) *In vitro* release profiles of cyclosporine from the contact lenses containing the free cyclosporine and the cyclosporine/C-HA micelles in PBS at pH 7.4 and 37 °C.

The drug loading efficiency of cyclosporine within the micelles was measured to be *ca.* 64% by comparing the absorbance of cyclosporine at 215 nm before and after drug loading (Fig. 5a). The *in vitro* drug release from the cyclosporine/C-HA micelle-embedded contact lens was analyzed in PBS at 37 °C. The release rate of cyclosporine was determined by the calibration curve of cyclosporine at 215 nm using HPLC analysis with a C18 column (ESI Fig. S6†). The recommended eye drop dosage for a 0.05% solution of cyclosporine is 2 drops per day. This means that the daily dose of cyclosporine is *ca.* 2 μg. The amount of cyclosporine loaded in the contact lens was determined by the above cyclosporine loading efficiency. After 12 days, approximately 50% of the loaded cyclosporine was continuously released from the contact lenses in comparison to the burst drug release from the control lens (Fig. 5b). The released amount of cyclosporine from the cyclosporine/C-HA micelle-embedded contact lens was 16 μg, which might be high enough for the therapeutic effect on dry eye syndrome for a week. The micelle-embedded contact lens might enable a longer time of delivery of cyclosporine compared to eye drops, resulting in higher drug delivery efficiency to the eye. The hydrophobic drugs loaded into the nanoparticles must first diffuse through the micellar structure in the contact lens hydrogel and then diffuse through the contact lens hydrogel. The longer release time of cyclosporine resulted in higher bioavailability of the drug, which contributed to a higher therapeutic effect on dry eye syndrome.

### 3.3 *In vivo* biological activity of cyclosporine/C-HA micelles

In order to investigate the therapeutic effect of cyclosporine/C-HA micelle-embedded contact lenses on dry eye syndrome, we tested three kinds of samples: the normal control contact lens, cyclosporine by eye drops (0.05 mg mL^−1^), and cyclosporine/C-HA micelle-embedded contact lens on the right eye (oculus dexter, OD) of DED model rabbits for a week. The left eye (oculus sinister, OS) was bare and did not have a contact lens. The patency was assessed by the Schirmer tear test, corneal fluorescein staining, and DED marker MMP9 analysis.

**Fig. 6:**
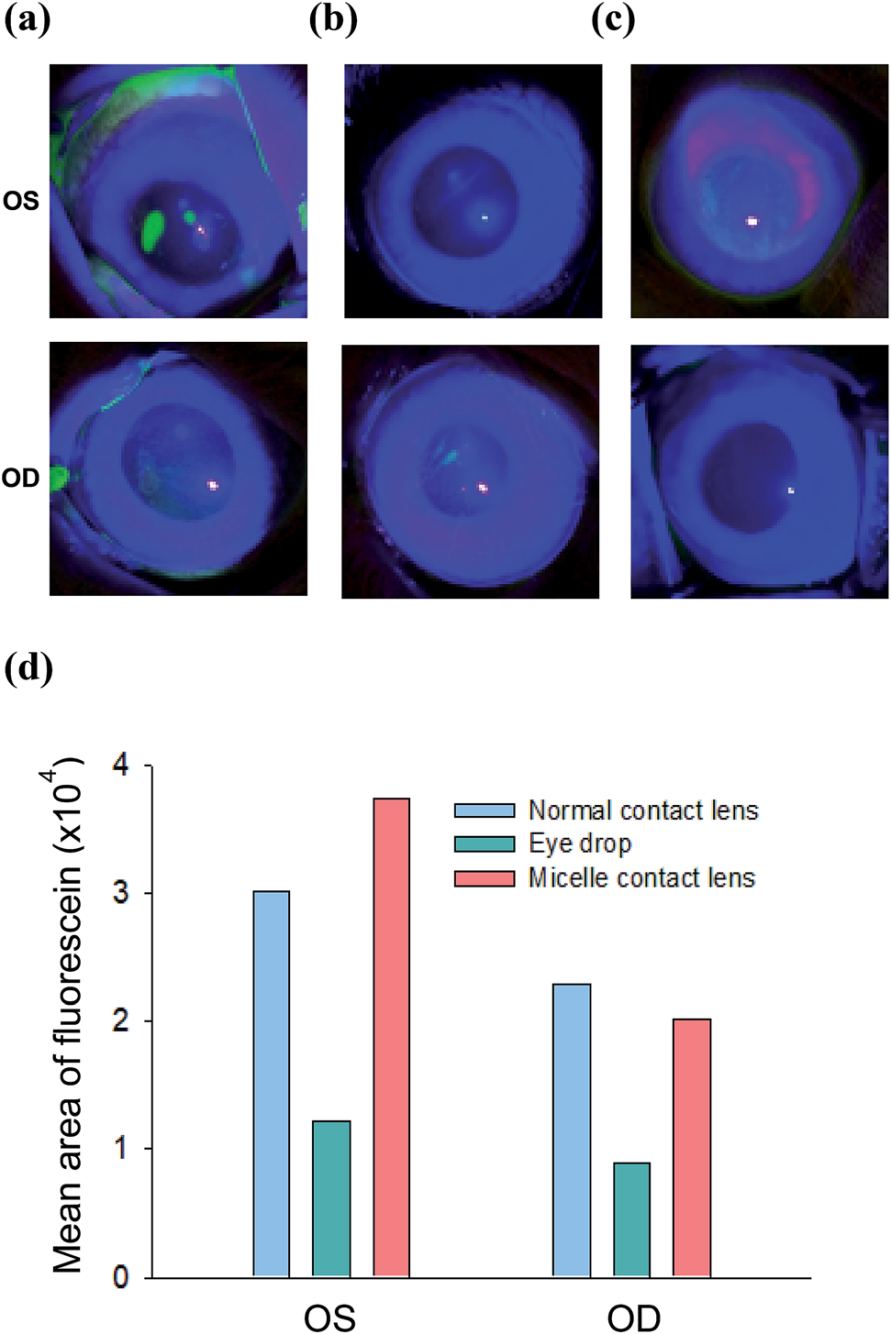
Corneal fluorescein staining for the analysis of corneal inflammation after (a) wearing the normal contact lens, (b) administration of cyclosporine by eye drops, and (c) wearing cyclosporine/C-HA micelle-embedded contact lens. (d) The ROI values of the fluorescein staining.

Fig. 6a shows the image of corneal fluorescein staining for each group. For fluorescein staining, sodium fluorescein was topically applied to the ocular surface of rabbits, typically without sedation. Several minutes after the treatment, corneal fluorescein staining was scored under a microscope using a blue light for the irregularities in the eye, such as abrasion and inflammation, which fluoresce with a great intensity relative to that of the healthy corneal tissue. In the case of ODs with the control normal contact lens and cyclosporine eye drops, the fluorescence signal remained after wearing normal contact lens or treatment by eye drops for 7 days. In contrast, OD with the cyclosporine/C-HA micelle-embedded contact lens showed significantly low fluorescence intensity, reflecting that the corneal surface inflammation was greatly reduced; this is possibly due to the therapeutic effect of the released cyclosporine. Fig. 6d shows the quantitative fluorescence analysis in pixel units.

**Fig. 7:**
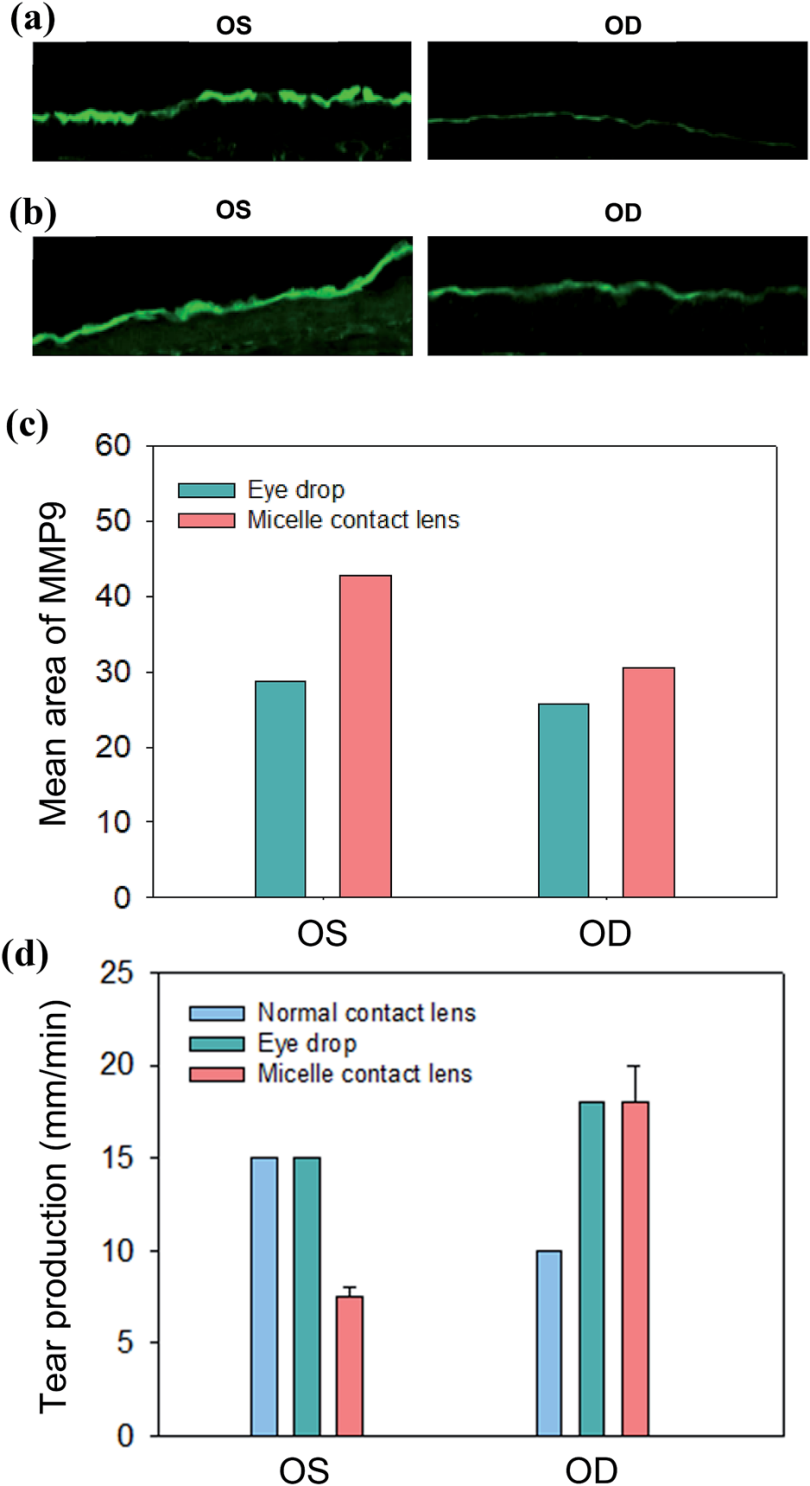
Corneal inflammation analysis by the corneal fluorescein staining of DED marker of MMP9 after (a) cyclosporine eye drops and (b) wearing cyclosporine/C-HA micelle-embedded contact lens. (c) The ROI values for the MMP9 analysis. (d) The tear production of the control eye (OS) and the eye wearing the contact lens (OD) at day 7: wearing the normal contact lens (sky blue), cyclosporine eye drops (green), and wearing the cyclosporine/C-HA micelle-embedded contact lens (pink, *n* = 2).

The immunofluorescence staining analysis for a dry eye marker of MMP9 confirmed the therapeutic effect of the cyclosporine/C-HA micelle-embedded contact lens treatment. Fig. 7a and b show the MMP9 staining in the cornea. The intensity of MMP9 was slightly reduced in the eye treated by cyclosporine eye drops. In the case of rabbits wearing the cyclosporine/C-HA micelle-embedded contact lens, significant reduction of MMP9 was observed in the cornea (Fig. 7c). The results indicated that cyclosporine was released from the C-HA micelle-embedded contact lens and caused a significant therapeutic effect on dry eye syndrome.

Fig. 7d shows the tear production of eyes wearing the cyclosporine/C-HA micelle-embedded contact lens for 4 h a day in comparison to that without wearing the contact lens. In the case of cyclosporine eye drops, two drops were administered per day. The analysis of tear production after 7 days showed decrease in rabbits wearing the normal contact lens and slight increase in rabbits treated by cyclosporine eye drops. In contrast, the tear production was greatly enhanced for the rabbits wearing the cyclosporine/C-HA micelle-embedded contact lens, suggesting that the drug was steadily released from the contact lens, which was sufficient for the therapeutic effect.

## 4. Conclusions

C-HA was successfully synthesized by conjugating CAEC to HA-TBA in DMSO by amide bond formation between the amine groups in CAEC and the carboxyl groups in HA. Cyclosporine as a dry eye therapeutic drug was loaded in the micellar structure of the synthesized C-HA. The formation of cyclosporine-loaded micelles was confirmed by DLS, zeta potential, TEM, and HPLC analyses. The transmittance analysis, EWC test, WE test, water contact angle analysis, and tensile strength test confirmed the improved physical and mechanical properties of C-HA micelle-embedded contact lenses compared to those of the normal PHEMA contact lens as a control. *In vitro* release tests showed the continuous release of cyclosporine from the cyclosporine/C-HA micelle-embedded contact lens for more than 10 days. Finally, the therapeutic effect on dry eye syndrome was successfully confirmed by the Schirmer tear test, corneal fluorescein staining, and MMP9 fluorescein analysis in the DED model rabbits. Taken together, we can confirm the feasibility of cyclosporine/C-HA micelle-embedded contact lenses for further clinical development. These drug-eluting contact lenses would be greatly beneficial for dry eye syndrome patients wearing contact lenses.

## Conflicts of interest

There are no conflicts to declare.

## Supplementary Material

RA-009-C9RA02858G-s001
