# Supplementary material for: Drug-eluting contact lens containing cyclosporine-loaded cholesterol-hyaluronate micelles for dry eye syndrome
Source: RSC Adv. 2019 May 28;9(29):16578–85. doi: 10.1039/c9ra02858g (PMC9064448; doi:10.1039/c9ra02858g)
Supplement: RA-009-C9RA02858G-s001 [file RA-009-C9RA02858G-s001.pdf]

Electronic Supplementary Material (ESI) for RSC Advances.
This journal is © The Royal Society of Chemistry 2019

Electronic Supplementary Material (ESI) for RSC Advances.
This journal is © The Royal Society of Chemistry 2019

Electronic Supplementary Information (ESI)

Drug Eluting Contact Lens Containing Cyclosporine Loaded Cholesterol-Hyaluronate Micelle for Dry Eye Syndrome

Jonghwan Mun,† Jee won Mok,‡ Sanghoon Jeong,† Seonghwi Cho,† Choun-Ki Joo,‡,* Sei Kwang Hahn†,*

† Department of Materials Science and Engineering, Pohang University of Science and Technology (POSTECH), 77 Cheongam-ro, Nam-gu, Pohang, Gyeongbuk 37673, Korea.

‡ Department of Ophthalmology and Visual Science, Seoul St. Mary's Hospital, Collage of Medicine, The Catholic University of Korea, 505, Banpo-dong, Seocho-gu, Seoul 06591, Korea.

***Corresponding authors**

Tel.: +82 54 279 2159. Fax: +82 54 279 2399. E-mail: skhanb@postech.ac.kr (S.K. Hahn)

Tel: +82 2 2258 1188. Fax: +82 2 599 7405. E-mail: ckjoo@catholic.ac.kr (C.K. Joo)

This journal is © The Royal Society of Chemistry 2019

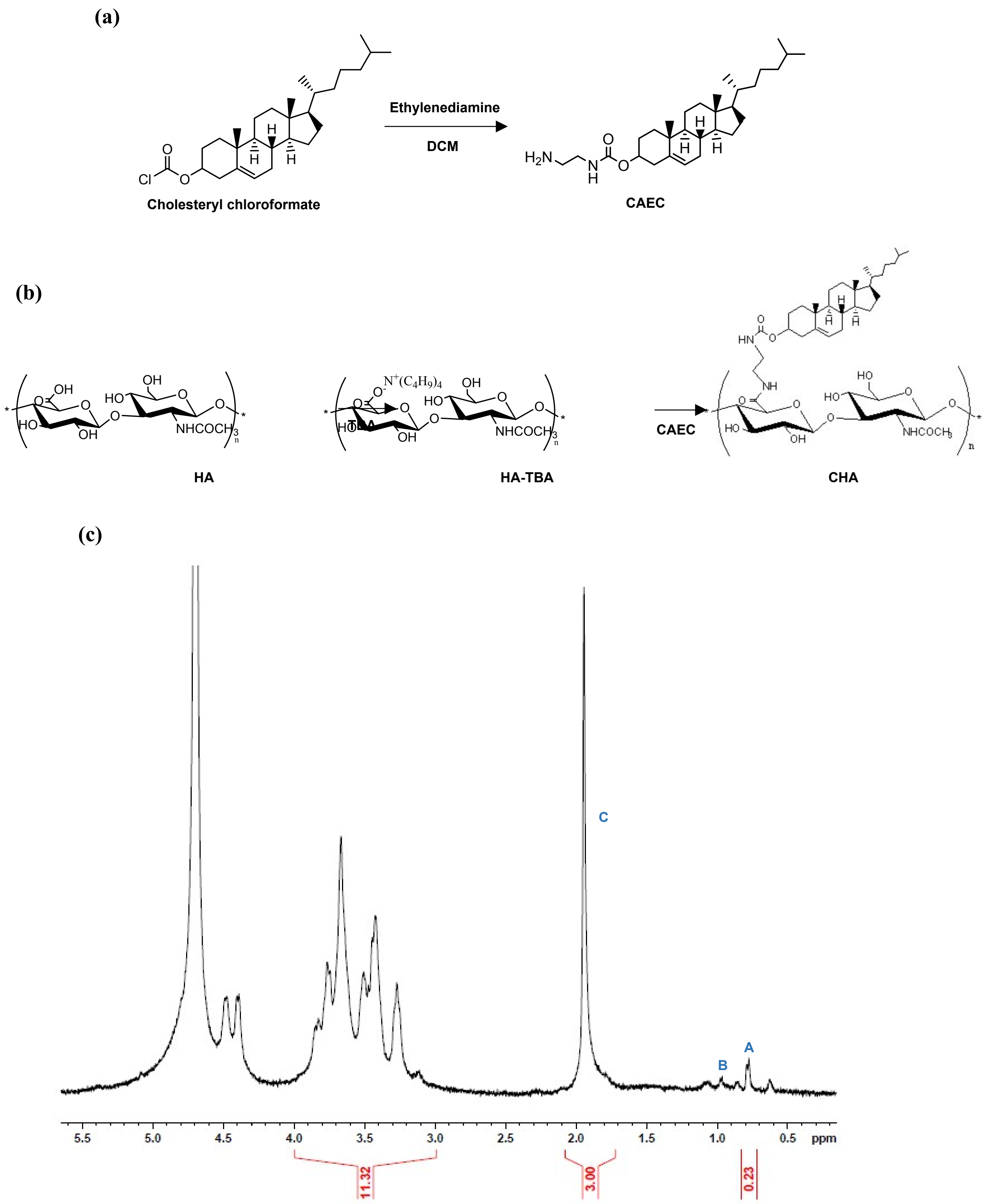

Figure S1. Synthetic scheme for (a) CAEC and (b) C-HA. (c) ^{1}H NMR data for C-HA.

This journal is © The Royal Society of Chemistry 2019

(a)

(b)

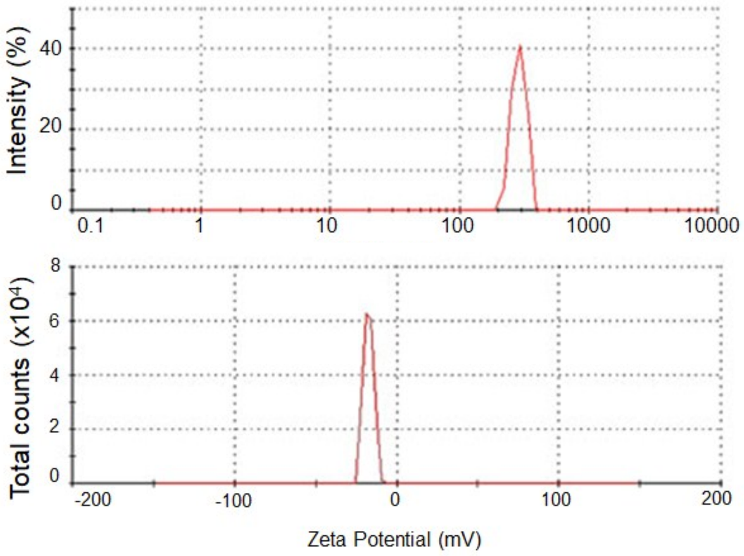

Figure S2. (a) Particle size and (b) zeta potential analyses of cyclosporine loaded C-HA micelle by dynamic light scattering.

This journal is © The Royal Society of Chemistry 2019

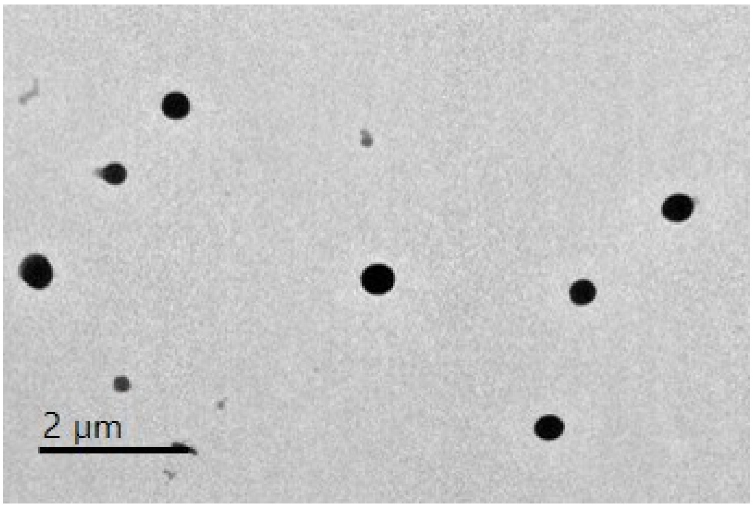

Figure S3. TEM image of cyclosporine loaded C-HA micelles.

This journal is © The Royal Society of Chemistry 2019

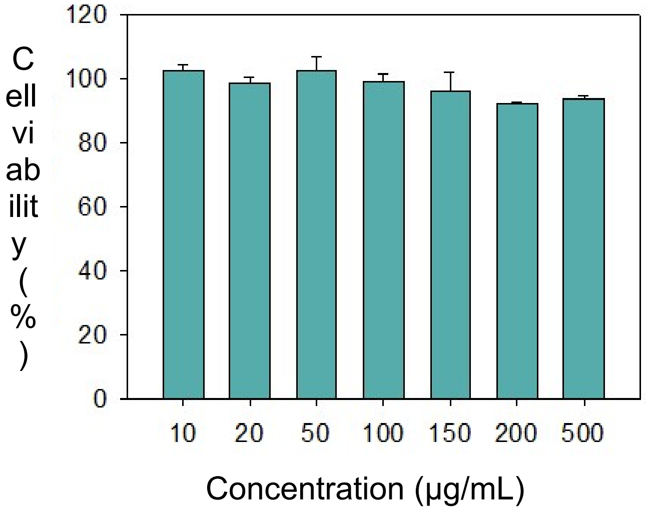

Figure S4. The cytotoxicity of C-HA micelles with increasing concentrations from 10 to 500 μg/mL in human cornea epithelial cells (n = 5).

(a)

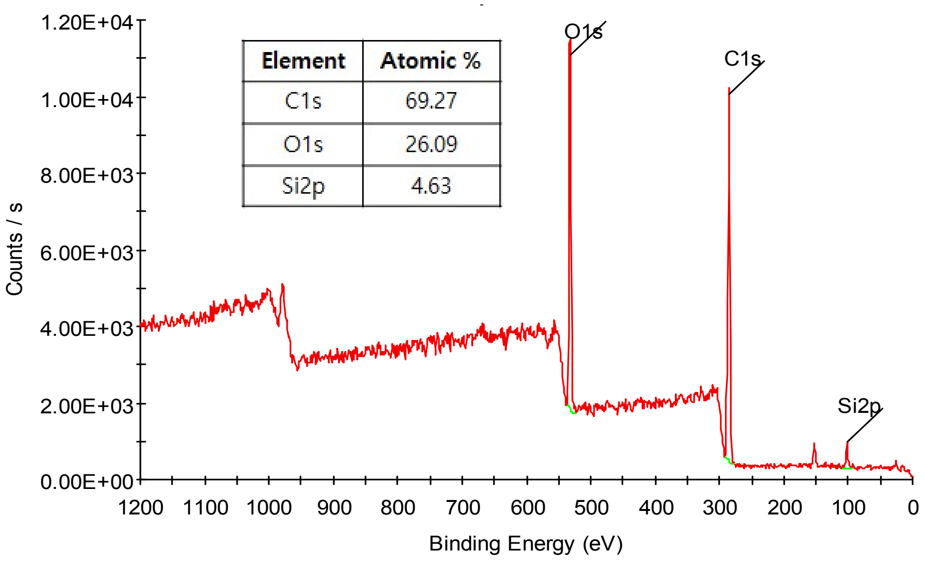

(b)

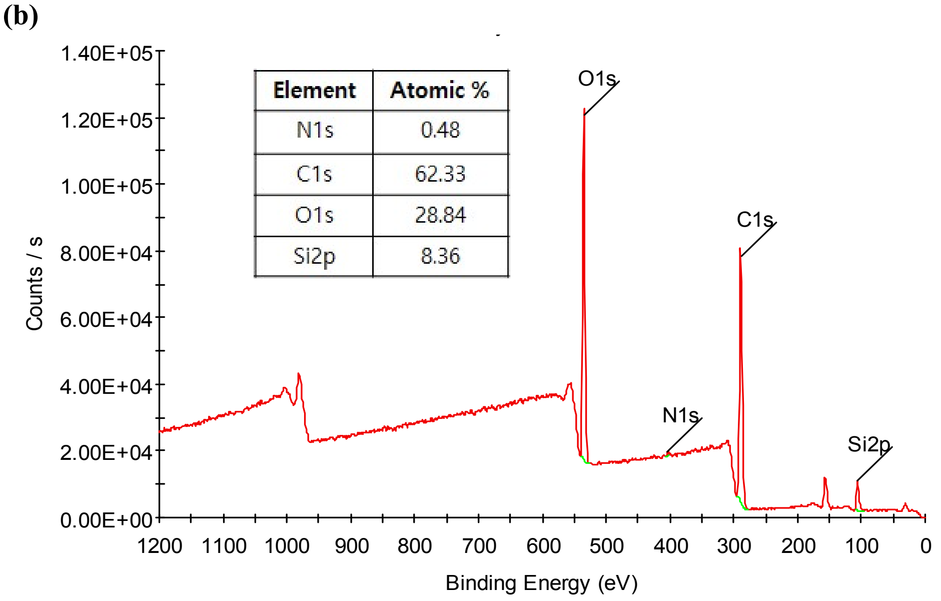

Figure S5. XPS analysis of (a) the normal contact lens and (b) the micelle contact lens.

This journal is © The Royal Society of Chemistry 2019

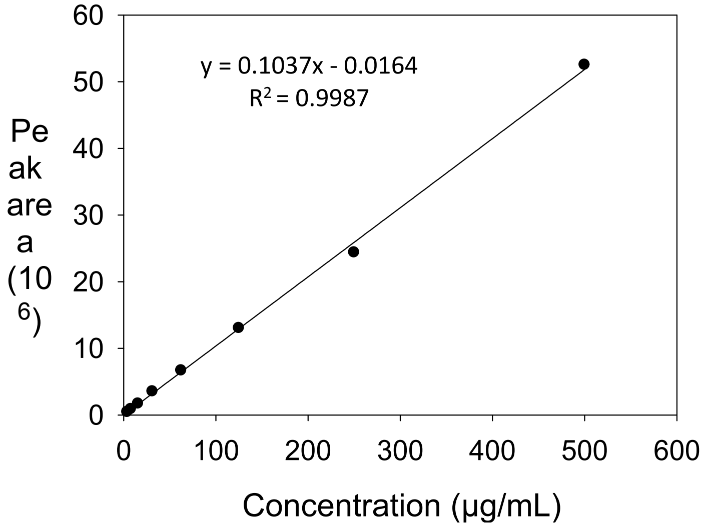

Figure S6. The calibration curve of cyclosporine at 215 nm by HPLC.
